# Advances in the Field of Two-Dimensional Crystal-Based Photodetectors

**DOI:** 10.3390/nano13081379

**Published:** 2023-04-15

**Authors:** Xiaoling Ye, Yining Du, Mingyang Wang, Benqing Liu, Jiangwei Liu, Syed Hassan Mujtaba Jafri, Wencheng Liu, Raffaello Papadakis, Xiaoxiao Zheng, Hu Li

**Affiliations:** 1 Shandong Technology Centre of Nanodevices and Integration, School of Microelectronics, Shandong University, Jinan 250101, China; 202112357@mail.sdu.edu.cn (X.Y.); 202000400094@mail.sdu.edu.cn (Y.D.); mingyangwang@mail.sdu.edu.cn (M.W.); 202132396@mail.sdu.edu.cn (B.L.); 201912222@mail.sdu.edu.cn (W.L.); 2 School of Energy and Power Engineering, Shandong University, Jinan 250061, China; jiangwei.liu@sdu.edu.cn; 3 Department of Electrical Engineering, Mirpur University of Science and Technology (MUST), Mirpur Azad Jammu and Kashmir 10250, Pakistan; hassan.jafri@must.edu.pk; 4 Department of Chemistry, Uppsala University, 75120 Uppsala, Sweden; rafpapadakis@gmail.com; 5 TdB Labs AB, Uppsala Business Park, 75450 Uppsala, Sweden; 6 Shenzhen Research Institute of Shandong University, Shenzhen 518057, China; 7 Department of Materials Science and Engineering, Uppsala University, 75121 Uppsala, Sweden

**Keywords:** two-dimensional material, optical properties, graphene, photodetector

## Abstract

Two-dimensional (2D) materials have sparked intense interest among the scientific community owing to their extraordinary mechanical, optical, electronic, and thermal properties. In particular, the outstanding electronic and optical properties of 2D materials make them show great application potential in high-performance photodetectors (PDs), which can be applied in many fields such as high-frequency communication, novel biomedical imaging, national security, and so on. Here, the recent research progress of PDs based on 2D materials including graphene, transition metal carbides, transition-metal dichalcogenides, black phosphorus, and hexagonal boron nitride is comprehensively and systematically reviewed. First, the primary detection mechanism of 2D material-based PDs is introduced. Second, the structure and optical properties of 2D materials, as well as their applications in PDs, are heavily discussed. Finally, the opportunities and challenges of 2D material-based PDs are summarized and prospected. This review will provide a reference for the further application of 2D crystal-based PDs.

## 1. Introduction

Two-dimensional (2D) materials have received more and more attention in recent years. This family of materials developed rapidly, and their unique structures endow them with many excellent properties that allow them to be promising candidates for the next generation of optoelectronic devices, such as high-performance photodetectors (PDs). First, the quantum confinement in the direction perpendicular to the 2D plane enables 2D materials to acquire exceptional electrical and optical properties, which are favorable for light absorption, ultrafast, and ultrasensitive photodetection [1]. Second, the surface of 2D materials is naturally passivated and lacks dangling bonds, making them easy to integrate with silicon chips [1,2]. Third, due to their diverse electrical characteristics, 2D materials can cover a wide range of electromagnetic spectrum responses [3,4]. Finally, some 2D materials with atomically thin characteristics, such as graphene and black phosphorus (BP), can construct scale-to-nano devices free of parasitic capacitors [5] because their photoelectric properties can be altered by local fields [6,7,8]. In addition, it is possible to build vertical heterostructures using distinct 2D materials without the “lattice mismatch” issue produced by Van der Waals interactions.

**Figure 1:**
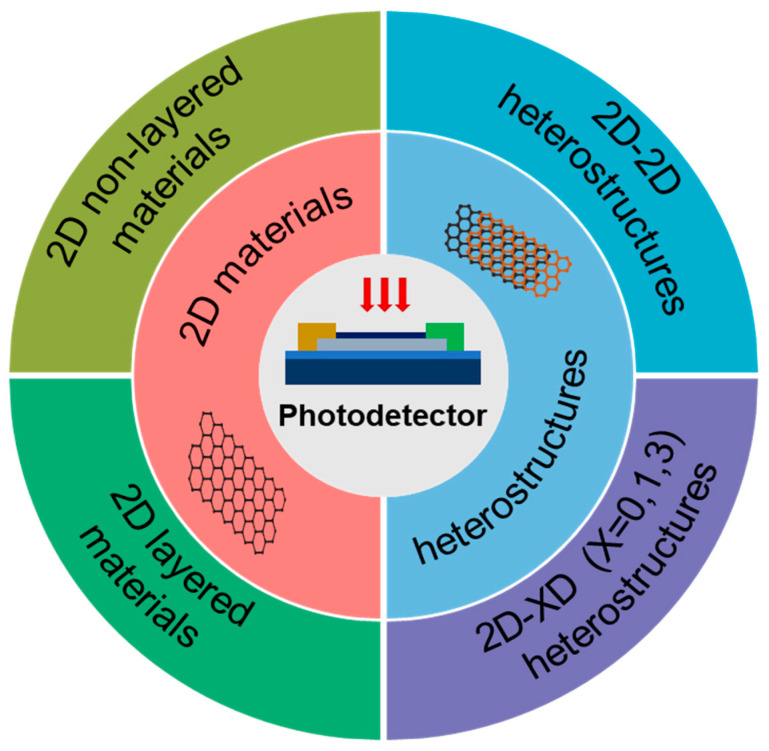
The research focus of 2D materials for PDs, including 2D nonlayered materials, 2D layered materials, and their heterostructures.

Benefiting from these excellent properties, 2D materials, as well as the heterostructures, show great application potential in the fabrication of PDs (Figure 1) [9]. According to the different properties of each 2D material, they can be applied in different detection areas. For example, the zero bandgap and ultra-high carrier mobility of graphene can be used in the applications of photovoltaic cells, high-speed PDs, and photosensitive transistors [10,11,12]. In addition, its identical absorbance throughout a wide electromagnetic spectrum range offers it great application potential in broadband irradiation detection from ultraviolet to terahertz (THz) [13]. In addition to graphene, BP, transition-metal dichalcogenides (TMDs), and hexagonal boron nitride (hBN) have also become research hotspots. The unique properties of single layer BP, called “phosphorene”, allow it to show great potential in optoelectronics applications [14]. BP is a direct bandgap semiconductor, and its electron–hole pairs can be easily excited by absorbing visible or near-infrared (NIR) light [15]. Similar to BP, by adjusting the number of layers of TMDs, the bandgap can be tuned in the range of 1.1~1.9 eV, leading to their wide applications in the field of optoelectronics. hBN with a graphite-like layered structure is also an attractive 2D material [16]. Since charge carriers driven by high-energy electrons or photons can efficiently emit light at deep ultraviolet (DUV) frequencies via strong electron–phonon interactions, hBN has the potential to be utilized in UV light-emitting devices.

2D materials have broad application prospects in optoelectronics devices, and they hold the promise to break through the development bottleneck of conventional PDs [17,18,19,20]. In this review, we provide a systematic survey of recent progresses in 2D material-based PDs. First, we briefly introduce the light detection mechanism of PDs based on 2D materials. Subsequently, we introduce the structure and optical properties of various 2D materials and summarize the recent development of corresponding PDs. Lastly, the challenges and perspectives of 2D material-based PDs are prospected. This review may provide a deeper grasp of 2D materials, offering insights in device fabrication for commercialization.

## 2. Photocurrent Generation Mechanism

PDs are crucial functional devices capable of converting illusive optical impulses into electrical signals, which are essential for numerous applications [21,22]. The basic working mechanism includes: (1) photogenerated charge carriers generated by illumination; (2) current generated by carrier diffusion or drift; (3) amplification of the photocurrent and transformation of voltage signals in the amplifier circuit. According to the nature of physical effects caused by incident radiation, PDs can be divided into two types. One is photon detectors based on the photoelectric effect. The other is thermal detectors that rely on changes in electron or lattice temperature to cause the thermalization of carriers. The most notable characteristic of a thermal detector is that it is insensitive to the wavelength of light emission. 

### 2.1. Photonic-Type Mechanism

#### 2.1.1. Photovoltaic Effect

**Figure 2:**
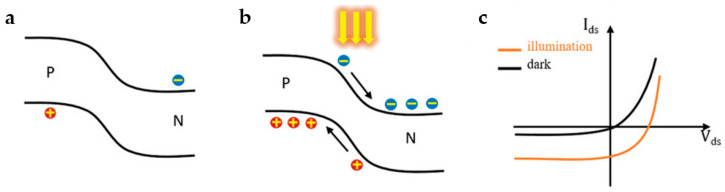
Schematic band diagrams of the *p*-*n* junctions (**a**) without illumination and (**b**) under illumination. (**c**) I–V characteristic of a *p*-*n* junction with and without illumination. (Reprinted with permission from ref. [9]. Copyright 2021 John Wiley and Sons Publications).

Numerous investigations have demonstrated that the core of a photoelectric effect is the built-in electrical field, which drives the separation of charge carriers created by illumination, to a certain extent. The built-in electrical field is realized at the semiconductor–metal interface using chemical doping, heterostructure manufacturing, and other techniques. Taking the *p*-*n* junction as an example, the concentration gradients of electrons and holes in the *p*-*n* junction force them to move relative to each other, resulting in a built-in electrical field at the interface, as illustrated in Figure 2a. Under the influence of a built-in electrical field, the electron–hole pairs generated by illumination are separated to form photocurrents, whose flow direction is related to the orientation of the electrical field (Figure 2b). This phenomenon is generally known as the photovoltaic (PV) effect, which can facilitate the separation of photo-excited carriers. Figure 2c shows the nonlinear I–V characteristic curves of a *p*-*n* junction, which exhibit the rectification feature in both light and dark conditions [23]. Under illumination with a bias voltage (V_ds_) of 0 volts, there is an appreciable photogenerated current (I_ds_). When the energy of a photon exceeds the bandgap, the electron–hole pairs are excited and then form short circuit currents after being separated by the built-in electrical field. If the circuit is open, the holes and electrons accumulate in the device to form an open-circuit voltage. Assuming that a sizeable reverse bias is applied to a heavily doped *p*-*n* junction, the incident light will accelerate the generation of charge carriers, resulting in a high photoconductive gain in PDs [5]. Due to the electrical field generated by the *p*-*n* junction’s inherent potential, the carriers’ lifetime can also be extended. Self-powered PDs are typically based on PV effects in semiconductors because detecting zero-bias photons requires less power consumption, low light noise caused by dark current, and an exceptionally high photocurrent to dark current ratio [24,25,26,27].

#### 2.1.2. Photoconductive Effect

**Figure 3:**
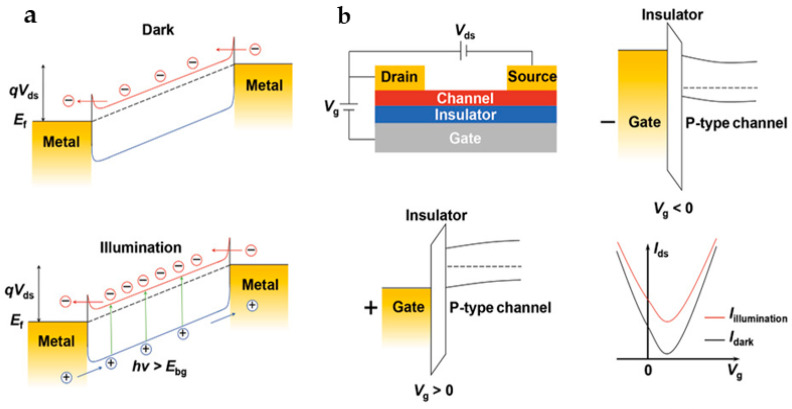
(**a**) A schematic diagram of the PC effect mechanism. Under dark conditions (upper panel), a semiconductor channel contacted by two metal electrodes produces a dark current I_dark_ at a bias voltage of V_ds_. Under illumination (bottom panel), the extra carriers excited by incident photons lead to a large photocurrent I_light_ at a bias voltage of V_ds_. (**b**) The principle diagram of a back-gated PD (top-left panel), the band diagram of the gate/insulator/channel of the PD under the dark conditions at a negative gate bias (top-right panel) and at a positive gate bias (bottom-left panel), and the relationship between the source-drain current and gate bias under illumination and dark conditions (bottom-right panel). (Reprinted with permission from ref. [30]. Copyright 2021 John Wiley and Sons Publications).

The photoconductance (PC) effect employs the mechanism by which surplus charge carriers are activated when semiconductors absorb photons, resulting in an increase in material conductivity and a decrease in resistance (Figure 3) [28]. As shown in Figure 3a, in the darkness, the absorbed free carriers are driven by the applied V_ds_ to produce a tiny dark current (I_dark_). However, under illumination, when the photon energy exceeds the semiconductor’s bandgap, the electrons in the valence band will jump to the conduction band, forming electron–hole pairs. Electron–hole pairs separated by V_ds_ drift opposite to the source-drain stage, thus generating currents (I_ligh_) that are more significant than dark currents. The net current increase is called photocurrent (I_photo_), which can be represented as I_photo_ = I_light_ − I_dark_. In general, most carriers have superior mobility at moderate V_ds_, thus having short migration periods [29]. The lifetime of photogenerated carriers substantially impacts the response speed and photoconductive gain. Figure 3b depicts a PD employing the PC effect, which consists of a semiconductor channel and two ohmic contacts connected to the ends of the channel as source-drain poles. The photo-field effect transistor (photo-FET) is a famous structure of PD that typically utilizes a back-gated configuration (Figure 3b) to prevent incident light from being obstructed by the electrode. Photo-FETs using 2D channel materials have an advantage over bulk materials in achieving lower dark currents at the same gate bias because the depletion region can extend through the entire 2D channel thickness, allowing for better current control and improving efficiency [30]. The band diagram of the gate/insulator/channel of PDs at negative gate bias (top-right panel) and positive gate bias (bottom-left panel) is shown in Figure 3b. In order to achieve a high photoconductive gain in PDs, it is important to either increase the lifetime of the photogenerated carriers or decrease the transit time of electrons. However, a longer lifetime of photocarriers leads to a slower recombination process, which can negatively impact the response speed of the PDs. Therefore, it is crucial to strike a balance between gain and response speed by managing this trade-off effectively. The bottom-right panel demonstrates the relationship between the source-drain current and the gate bias under dark and illuminated conditions. Compared with the dark condition, the source-drain current versus gate bias curve shows a shift upwards under illumination conduction, indicating a higher level of conductivity in the channel region due to the generation of electron–hole pairs by incident light. The magnitude of this shift can be used to detect and quantify the intensity of incident light, making PDs an important component in various applications such as imaging and sensing.

#### 2.1.3. Photogating Effect

**Figure 4:**
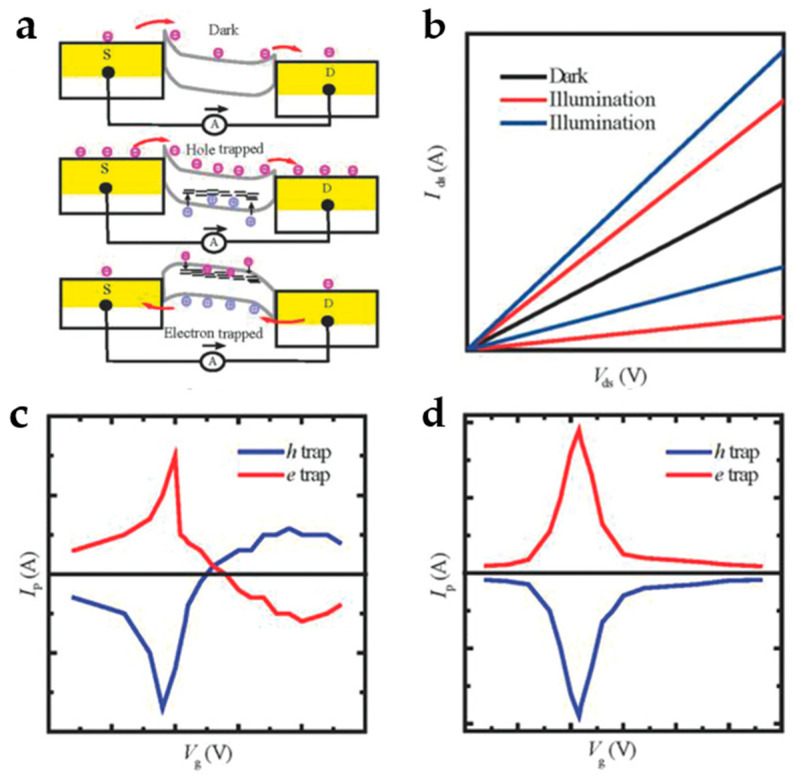
(**a**) Schematic of PG effect in the dark state in the upper panel, hole trap in the middle panel, and electron trap in the bottom panel. (**b**) I–V curves in dark and illuminated states. (**c**,**d**) Photocurrent as a function of gate voltage for ambipolar and unipolar FET devices, respectively. (Reprinted with permission from ref. [5]. Copyright 2018 John Wiley and Sons Publications).

The photogating (PG) effect (Figure 4a) is a photocurrent generation process seen as a particular case of the PC effect that can produce significant responsiveness in PDs [31]. Due to numerous defects and traps in semiconductors, light-generated electrons and holes may be captured by localized states on the defects or surface (Figure 4a). If the carriers are trapped in the trap states, the charged trap states can act as a localized floating gate, influencing the channel conductance [32]. As a result, the conductivity of a channel can be tuned in this way. Because of the slow detrapping process, the photogenerated carriers in the semiconductor might undergo multiple cycles, thus resulting in a high gain. The I–V curves in darkness and illumination indicate that the photocurrent may be larger or smaller than the dark current (Figure 4b). For bipolar semiconductors, the photocurrent’s signal varies with the function of gate voltage (Figure 4c), whereas for unipolar semiconductors, the photocurrent is either larger or smaller than the dark current and is unaffected by gate voltage (Figure 4d). In bipolar FET devices, both electrons and holes contribute to the photocurrent, and the gate voltage can control the relative contributions of these two types of charge carriers. As a result, the photocurrent in bipolar FETs can be positive or negative depending on the gate voltage applied. In contrast, the photocurrent in a unipolar FET is generated by the same type of charge carriers (either electrons or holes) that are responsible for the device’s electrical conductivity. The gate voltage in unipolar FETs controls the flow of these charge carriers, but it does not change the type of charge carriers that are present in the device, as there is no junction in a unipolar device. Fukushima et al. constructed a medium IR graphene PD utilizing the PG effect in 2020 [33]. The PD consisted of a source drain, an insulating layer, a graphene channel on top, and a photosensitizer. Results indicated that the response to medium IR light with the PG effect was 100 times greater than that of conventional graphene detectors lacking the PG effect. The PD’s responsivity increased from 61.7 to 321.0 A/W when the channel area was reduced from 100 to 25 μm^2^.

### 2.2. Thermal-Type Mechanism

#### 2.2.1. Photothermoelectric Effect

The photothermoelectric (PTE) effect is a novel photocurrent mechanism that can convert energy between light, heat, and electricity without requiring an external electrical field to separate electron and hole pairs generated by photoexcitation. The PTE effect generates electrical energy via a temperature gradient formed by hot carrier diffusion in the device. When the light spot is smaller than the device’s channel size, a temperature difference (ΔT) arises across the semiconductor channel, driving the diffusion of electron holes to form a PTE voltage on the channel, which may be represented as V_PTE_ = SΔT [30] (S is the Seebeck coefficient of the semiconductor). It is reported that the PTE effect depends on the Seebeck coefficient fluctuation caused by the doping dispersion of 2D materials [34]. Furthermore, a second PTE effect, known as the PTE channel effect, may occur on the uniform graphene channel in the presence of electron temperature gradients. To date, the PTE effect has garnered significant attention because the heat energy lost during the relaxation process of photogenerated carriers can be exploited to improve the responsiveness of PDs and the energy conversion efficiency of solar cells. The reported PTE effect studies have concentrated on low-dimensional materials such as graphene, black phosphorus, III–V semiconductor nanowires, and so on. Nonetheless, the poor light absorption of these materials and the difficulty of large-area controllable preparation limit the practical application of the PTE effect as a new photoelectric conversion mechanism in solar cells and PDs.

#### 2.2.2. Photobolometric Effect

The Photobolometric (PB) effect refers to the temperature change of active materials after absorbing incident light under thermal radiation, which increases or decreases in resistivity [35,36]. Thus, at a fixed bias voltage, the device’s current will also change. The difference between the PB and PTE effects is whether or not an external bias is necessary for current conduction. Without an external bias, the photocurrent from the PTE effect can be self-driven, whereas this cannot be observed in the PB effect. Bolometers are photoelectric devices based on the PB effect that may be widely employed in the mid-IR to THz wavelength range due to the wavelength-independent features of the PB effect. Additionally, certain 2D material IR PDs also use the PB effect. In 2020, Xu et al. fabricated a SnSe IR PD based on the PB effect that attained a responsivity of 0.16 A/W under mid-IR light with a wavelength of 10.06 μm [37].

## 3. 2D Material-Based PDs

### 3.1. Graphene Based

In recent years, graphene-based materials, such as graphene nanoribbons, graphene oxide, and its reduced form, doped graphene materials, as well as other derivatives, have garnered significant interest in various scientific domains [38]. This is because they possess many intriguing qualities, including a vast surface area (approximately 2630 m^2^/g for a single layer of graphene), strong electrical conductivity, and unusual optical, thermal, and mechanical capabilities.

#### 3.1.1. Morphology and Structure

**Figure 5:**
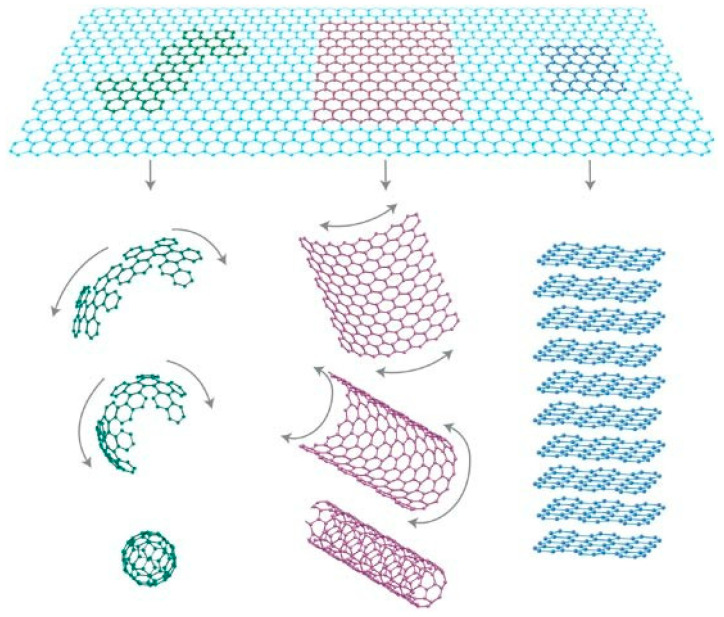
Schematic diagram of zero-dimensional fullerenes, one-dimensional carbon nanotubes, and three-dimensional graphite formed from graphene. (Reprinted with permission from ref. [44]. Copyright 2007 Springer Nature Publications).

In 2004, Novoselov et al. successfully exfoliated single-layer graphene from a graphite crystal sheet using the mechanical exfoliation approach, which challenged the scientific understanding of 2D crystals [39]. Graphene is a 2D carbon material with a honeycomb crystal structure (Figure 5). It is the thinnest and strongest nanomaterial, with a single layer thickness of 0.33 nm [40]. Each carbon atom in graphene is bonded to three neighboring carbon atoms via σ bonds, and the C–C bond length is only 0.142 nm [41]. In addition to σ bonds, graphene has a conjugated π-network that is structurally similar to an indefinitely large planar aromatic molecule. This feature gives graphene many excellent properties. The edge structure of graphene is similar to carbon nanotubes, which can be classified into zigzag and armchair forms based on different carbon chains with distinct transport properties. Graphene nanoribbons with zigzag edge patterns have spin-polarized capabilities, but those with armchair edge structures exhibit semiconducting properties [42]. Due to the existence of charge carriers, the electronic structure of few-layer graphene is complex. Some investigations have revealed that the electronic structure of graphene changes rapidly as the number of layers grows, approaching graphite’s 3D limit at ten layers [43]. Three-dimensional graphite is different from the traditional graphite, which has a layered two-dimensional structure, as it has a three-dimensional structure. By applying high pressure and high temperature on graphite, such as shock compression, 3D graphite can be obtained. The high pressure and temperature cause the graphite layers to deform and bond together, resulting in a 3D structure. In addition to shock compression, another method to produce 3D graphite is by using diamond anvil cells. As a result of the high pressure generated by compressing two diamond anvils, the layers of graphite deform and combine; then, a 3D structure can also be obtained. The distinct properties of 3D graphite make it a desirable material for various applications, such as serving as a high-capacity electrode material in batteries and as a component in high-strength materials used in the aerospace and defense industries.

#### 3.1.2. Optical Properties

Graphene has outstanding optical qualities. It has an absorptivity of roughly 2.3% over a wide wavelength range, as well as a transmittance constant of 97.3% for visible and IR light [45,46]. The optical properties of large surface area graphene films vary with the thickness, with the absorbance increasing by 2.3% for each additional layer. Furthermore, the graphene bandgap may be altered from 0 to 0.25 eV by supplying a voltage to the double-gate bilayer graphene FET at ambient temperature, and the optical response can be tuned to the THz region by applying a magnetic field [47]. In addition, graphene’s absorption will reach saturation when the incident light intensity surpasses a particular threshold value. Since graphene is a semi-metallic material, it exhibits ultra-high carrier mobility of up to 20,000 cm^2^/(Vs) at low temperatures [39]. The electron mobility of monolayer graphene is roughly 15,000 cm^2^/(Vs) [45,48] at temperatures ranging from 50 to 500 K, making it a suitable candidate for PDs.

#### 3.1.3. PDs Based on Graphene

**Figure 6:**
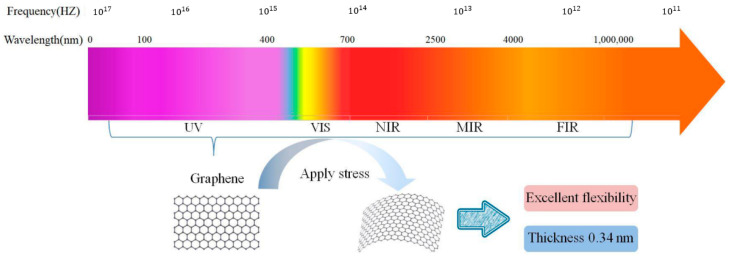
The photodetection range and properties of graphene. (Reprinted from ref. [49].).

Graphene can maximize the gain of PDs and has wide electromagnetic spectrum responses. Due to the zero bandgap and linear dispersion around the Dirac point, graphene can absorb light over a wide spectrum, making it possible to be used in the application of light detection in a wide spectral range (Figure 6) [49]. Moreover, graphene has excellent flexibility, which further expands its application in flexible optoelectronics. However, graphene’s poor light absorption, high noise, high dark current, and phototransistor’s complicated construction restrict its practical application. The first graphene PD was reported in 2009; Xia et al. demonstrated ultrafast PDs based on transistors made from single and multiple graphene layers [50]. Due to the exceptional electrical and photonic characteristics of graphene, the PD has an extraordinarily high bandwidth, zero source-drain bias, and excellent internal quantum efficiency. Since then, research on graphene-based PDs has primarily focused on enhancing their performance in terms of quantum efficiency, responsivity, and noise-equivalent power. The following will provide an overview of PDs that primarily used graphene over the last five years.

Graphene has potential applications in imaging devices considering its monolithic integration with complementary metal oxide semiconductors (CMOS) [51,52,53], strong field effects of electrostatic gates [54,55], and broadband absorption spectra [31,56]. Therefore, the incorporation of graphene into silicon-based image sensors can be employed to enhance sensitivity and spectral performance. Recently, Liu et al. reported a graphene charge-injection PD that combines the charge integration feature of the charge-coupled device with the CMOS’s independent pixel structure [57]. The detector exhibits high sensitivity (>0.1 A/W in the IR), high speed, broadband imaging (UV to mid-IR), high linearity, high fill factor, low noise, and low cost. In 2022, Ge et al. fabricated a flexible PD using 3D graphene films and organic materials, which achieved a high responsivity of 5.8 × 10^5^ A/W in the visible region and can detect light from visible to mid-IR at room temperature [58]. However, the photoresponsivity of graphene-based PDs is restricted to a few mA/W as a result of their ultra-fast hot carrier recombination characteristics and very poor light absorption [59]. The combination of graphene and 2D semiconductors may address the issue of a tiny effective junction area and simultaneously improve the light-quality interaction, but the semiconductor bandgap restricts the spectrum response range [60]. Additionally, the hybridization of graphene with quantum dots (QD) offers a reasonable and effective way to dramatically enhance the photoresponsivity of graphene-based PDs through a PG process [6]. It is reported that a PD based on graphene/SiO_2_/Si with an interface gating mechanism can detect a weak optical signal of 0.6 nW with an optical response rate of 1000 A/W at V_ds_ = 1 V [61]. In 2021, Huang et al. fabricated a graphene/HfO_2_/a-MOS_2_ PD that could detect light in the range of 473~2712 nm at room temperature, with a response time of 68 μs and a responsivity of 5.36 A/W [62]. In addition, the simple production process and low cost indicate the applicability of gated graphene PDs in the photoelectric area. Table 1 lists some reports on graphene-based PDs.

### 3.2. Transition Metal Carbides Based PDs

Recently, transition metal carbides, nitrides, and carbonitrides (MXenes) have aroused wide attention due to their excellent properties. MXenes have the typical formula M_n+1_X_n_T_x_ (*n* = 1, 2, 3), where M stands for an early transition metal such as Sc, Ti, V, Zr, Nb, Cr, or Mo; X is carbon or nitrogen; and T_x_ represents a surface functional group (such as –O, –OH, –F, etc.) [76,77]. They are typically made by selectively etching the A element in the MAX phase employing high concentrations of hydrofluoric acid, where A is the third or fourth main group element in the periodic table. In the last decade, MXene’s unique shape and structure, exceptional mechanical capabilities, and considerable carrier mobility have attracted the curiosity of researchers. Notably, the electrical properties and carrier transport qualities of MXenes can be modified by varying the surface functional group types [78,79].

#### 3.2.1. Morphology and Structure

**Figure 7:**
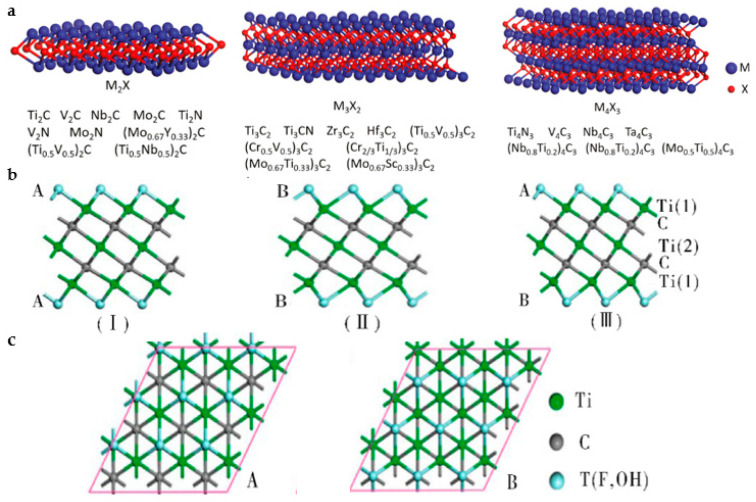
(**a**) X elements are interspersed between the second, third, and fourth layers of the M elements of M_2_X, M_3_X_2_, and M_4_X_3_, respectively. (Reprinted from ref. [80]). (**b**) Side view and (**c**) top view of Ti_3_C_2_T_2_ structure. (Reprinted with permission from ref. [83]. Copyright 2012 American Chemical Society Publications).

MXene’s hexagonal structure is inherited from its precursor MAX phase, which belongs to the space group P63/mmc. Figure 7a depicts the experimentally synthesized M_2_X, M_3_X_2_, and M_4_X_3_ structures [80]. In MXenes, *n* layers of X are covered by *n* + 1 layers of M, with van der Waals interactions connecting adjacent layers to form an [MX]_n_M arrangement [81]. Additionally, MXenes produced by etching with an acidic fluoride solution have intense surface activity and, thus, can easily form –OH, –O, and –F end groups by reacting with pollutants in solution. MXene offers three possible places for surface termination: (a) atop the transition metal atoms, (b) the position of the hole between the top metal atoms, and (c) the position of the hole between the next pile of X atoms [82]. The –O terminal site is more likely to occupy the vacancy site of the gap bond with the two metal atoms for stabilization because it requires two electrons to achieve a steady state, whereas the –OH and –F terminal sites require just one electron. Tang et al. outlined three MXene structure types based on the different orientations of –T (–F and –OH) in Ti_3_C_2_T_2_, as shown in Figure 7b,c [83]. The A-oriented T groups are positioned above the hollow sites between three nearby C atoms or point straight at the Ti (2) atoms, while the B-oriented T groups are positioned above the C atoms on the same side. Type I structures have orientation A on both sides; Type II structures have orientation B on both sides; and Type III structures have a mixed orientation, with orientation A on one side and orientation B on the other. Type I is the most structurally stable of the three, whereas Type II is the least stable. As a result, Type I is that in which the majority of MXenes are arranged. MXenes can be classified based on their M/X element ratio and crystal structure types, such as single M element type, solid solution M element type, ordered double M element type, etc. On the other hand, MXenes’ properties are intimately related to the M elements and T surface functional groups, which influence the majority of electronic and optical properties.

#### 3.2.2. Optical Properties

The band structure, including the energy bandgap, direct/indirect bandgap, etc., strongly influences the linear and nonlinear optical properties of MXenes. MXenes have potent light-absorbing abilities and can absorb light from UV to NIR. Ti_3_C_2_T_2_ films, for example, can absorb light from 300 to 500 nm. First, it is worth noting that the optical characteristics of MXenes rely on the number of layers and that transmittance rises with decreasing thickness, reaching >90% for layers with a thickness of 2.5 nm and <15% for layers with a thickness of 73 nm. Moreover, a significant transmission valley is detected between 750 and 800 nm as a result of surface plasmon resonance at 780 nm and the intrinsic transition between out-of-plane bands at around 800 nm [84]. Second, surface terminals formed during the experimental synthesis of MXenes impact its electronic structure and optical characteristics [85]. It was discovered that Ti_3_C_2_ with –F and –OH surface terminations had lower absorption coefficients than pristine Ti_3_C_2_ and Ti_3_C_2_ with –O terminations, making them suitable for transparent electrode applications [86]. The absorptivity and responsivity of Ti_2_CT_2_ (T = F, O, and OH) compounds in the IR to UV region have been shown to depend on the surface functional groups [87]. These findings suggest a possible way to modify the optical characteristics of MXenes by manipulating surface functional groups [88]. Finally, the optical properties of MXenes can be tuned by varying their chemical intercalation. For instance, the transmittance will change if different cations are intercalated into negatively charged Ti_3_C_2_T_x_ film layers. This behavior of changing transparency can be partially attributed to the change of c-axis lattice constant and charge transport [89].

#### 3.2.3. PDs Based on Mxenes

MXenes exhibit many intriguing characteristics, including excellent transparency, mechanical flexibility, high electrical conductivity, as well as a tunable work function that can be tuned by surface termination and internal composition. By utilizing their superior electronic and optical capabilities, they can serve as transparent conductive electrodes, Schottky contacts, conductive additives, light absorbers, charge transfer layers, and other essential roles in the application of optoelectronic devices. Among the current Mxene-related PDs, simple photoconductors, self-actuated PDs, and plasma-enhanced PDs have been reported. The following provides an overview of PDs that have primarily used MXenes over the last three years. Table 2 provides a summary of these PDs’ components, corresponding properties, and sources.

**Figure 8:**
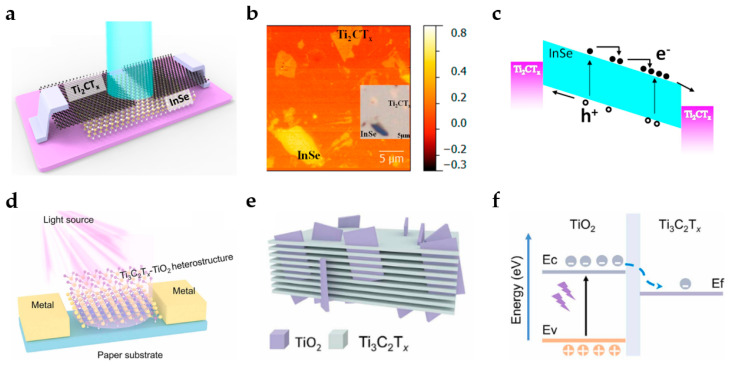
(**a**) Diagram of InSe/Ti_2_CT_x_ PD. Ti_2_CT_x_ is employed as the 2D electrode material. (**b**) Kelvin probe force microscopy potential mapping images showing InSe and Ti_2_CT_x_ slices. The inset figure shows the corresponding optical microscope image. (**c**) Illustration of the avalanche effect in an InSe/Ti_2_CT_x_ PD. (Reprinted with permission from ref. [90]. Copyright 2019 American Chemical Society Publications). Schematic diagram of (**d**) Ti_3_C_2_T_x_-TiO_2_ PD and (**e**) 3D-networked Ti_3_C_2_T_x_-TiO_2_. (**f**) Schematic band alignments and carrier flow at Ti_3_C_2_T_x_-TiO_2_ interfaces. (Reprinted with permission from ref. [91]. Copyright 2022 Elsevier Publications).

In addition to channel materials, electrodes are crucial PD components, and the usage of MXenes as high-performance PD electrodes has been documented in the scientific literature. Yang et al. created an InSe/Ti_2_CT_x_ PD (Figure 8a) in 2019 by designing a Ti_2_CT_x_ electrode as a nanoband array and analyzing the PD’s band structure with a Kelvin microscope (Figure 8b) [90]. The Fermi levels of InSe and Ti_2_CT_x_ are 4.4 eV and 4.9 eV, respectively. Due to the large work function of Ti_2_CT_x_, there is a sizeable Schottky barrier between InSe and Ti_2_CT_x_ suppressing the dark current and allowing a sufficiently high drain voltage to trigger an avalanche effect. Figure 8c depicts the band structure when the avalanche effect occurs, showing that a strong electrical field accelerates the photoexcited charge carrier to greater energy. Afterward, the carrier accumulates to generate additional pairs of hole electrons, resulting in a greater drain current. The performance of a PD is significantly enhanced by the avalanche effect, which shows a responsivity of 1 × 10^5^ A/W, a detectivity of 7.3 × 10^12^ Jones, and a dark current of 3 nA. Moreover, MXene has emerged as one of the alternative materials for transparent electrodes in the photonics research area, but it is challenging to create highly transparent and conductive MXene electrodes for flexible PDs. A translucent PD with excellent flexibility and photoresponsivity was created in 2020 by Chen et al. using a bio-inspired transparent MXene film with a light transmittance of roughly 90% and a resistance value of approximately 3 Ω/sq [104]. However, the practical application of pure MXenes in PDs is severely constrained by their high scattering rate and low light absorption, so it is necessary to investigate how heterojunctions affect these devices. Oxidized MXenes and MXene/perovskite nanocomposites have been identified as effective strategies for improving device performance because there is a synergistic interaction between the high carrier mobility of MXenes and the high absorption of the MXene/perovskite derivative [105,106]. In 2022, Xiong et al. established a 3D network Ti_3_C_2_T_x_-TiO_2_ PD using a controllable in situ oxidation method. The new PD demonstrated 13.3 times better performance than the original Ti_3_C_2_T_x_-based PD under 405 nm illumination, as illustrated in Figure 8d [89]. With morphologically controlled reagents, TiO_2_ nanosheets, a controllable oxidation derivative, were vertically inserted into layered Ti_3_C_2_T_x_ nanosheets to create Ti_3_C_2_T_x_-TiO_2_ heterostructures (Figure 8e). Ti_3_C_2_T_x_ nanosheets have superior photoelectric properties and light-absorbing capacity due to the presence of TiO_2_ derivatives, as demonstrated in Figure 8f. Subsequently, Ma et al. created a self-powered UV PD based on a Ti_3_C_2_T_x_/TiO_2_ heterojunction, which provides a high responsivity of 2.06 mA/W, short rise and decay times, and long-term stability [93].

### 3.3. Transition Metal Dichalcogenide (TMD) Based PDs

TMDs are layered semiconductor materials of the MX_2_-type, where M represents transition metal atoms (such as Mo, Re, W, Ta, etc.) and X is a chalcogen atom (such as S, Se, and Te). TMDs are appealing for optoelectronic applications due to their semiconducting properties and superior thermal stability [107,108,109]. There has been a significant amount of study conducted on TMDs. In 1923, Linus Pauling and others established the structure model of TMDs [110]. By the 1960s, over sixty varieties of TMDs had been identified [111]. The initial synthesis of monolayer MoS_2_ occurred in 1986, furthering the research on 2D-TMDs [112]. To date, the most reported TMDs are molybdenum disulfide (MoS_2_) [113], tungsten disulfide (WS_2_) [114], vanadium disulfide (VS_2_) [115], tungsten selenide (WSe_2_) [116], and molybdenum selenide (MoSe_2_) [117].

#### 3.3.1. Morphology and Structure

**Figure 9:**
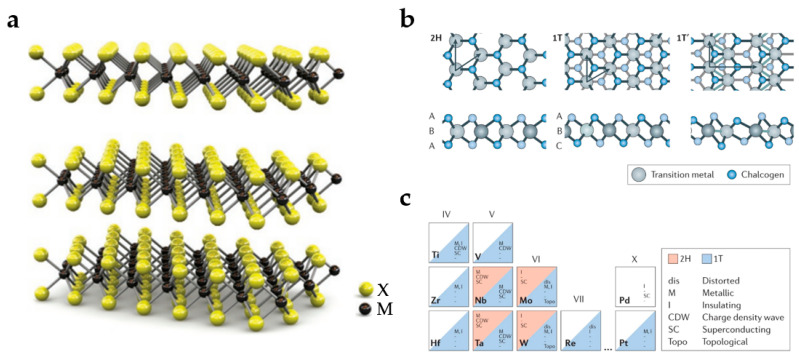
(**a**) The structure of TMDs. (Reprinted with permission from ref. [112]. Copyright 2011 Springer Nature Publications). (**b**) Atomic structures of monolayer TMDs in their triangular prismatic (2H), twisted octahedral (1T), and dimeric (1T′) phases. (Reprinted with permission from ref. [120]. Copyright 2017 Springer Nature Publications). (**c**) The existing structural phase of the TMDs (2H, 1T, or other), as well as the twisted structural phase and the observed electronic phase. (Reprinted with permission from ref. [120]. Copyright 2017 Springer Nature Publications).

As illustrated in Figure 9a, TMDs have a sandwich structure distinct from graphene, with chalcogen atoms placed in two hexagonal planes separated by a metal atom plane [118]. The atoms in these three layers are bound together by covalent bonds, and each layer is coupled by a weak Van der Waals force. Thus, the layers can be separated from each other [119]. On account of the distinct coordination modes of transition metal atoms, TMDs have a variety of structural phases. Triangular prism (2H) and octahedral (1T) coordination modes are the most common phases (Figure 9b) [120]. The various structures of monolayer TMDs can be viewed as the different stacking orders of the three atomic plane layers (chalcogene-metal-chalcogene elements) that comprise each layer of these materials. The 2H phases correspond to the stacking mode of ABA, in which the chalcogens in various atomic layers always occupy the same position A, and each chalcogen is just above the lower chalcogens in the direction perpendicular to the layer. Additionally, the 1T phases correspond to the ABC stacking order. The thermodynamically stable phase in the coordination of transition metals (groups IV, V, VI, VII, IX, and X) with chalcogenides (S, Se, and Te) is the 2H or 1T phase. Figure 9c summarizes the present understanding of the existence of two phases (stable or metastable forms) and other characteristics of TMDs. In group VI transition metals, the 2H phase is thermodynamically stable, whereas the 1T phase is metastable, with the exception of WTe_2_, whose stable phase at room temperature is the orthogonal 1Td phase [120]. For multilayer and bulk TMDs, the structure is typically described by the stacked structure of monolayer TMDs due to the possibility of periodicity-reducing distortions. If these distortions are extremely severe, they generate metal–metal bonds, which may transform the 1T structural phase of group VI TMD into the dimeric (1T′) phase. However, If the lattice distortion is weak, it leads to the formation of the charge density wave phase. Studies have shown that the thickness of TMDs can change their electrical and structural properties, which makes it easier to use them in micro- and nano-electronic devices. MoS_2_, one of the earliest members of TMDs, rose to prominence in 1963 after it was peeled off in an ultra-thin form utilizing tape technology, and then in a single layer in 1986. The fundamental structure of MoS_2_ consists of a single Mo atom sandwiched by two sulfur ions, comparable to the basic structure of graphene. The neighboring lattice spaces in MoS_2_ are occupied by various atoms that are related via spin-orbit coupling. The special structure of MoS_2_ endows it with attractive photoelectric properties, such as a direct bandgap of 1.8 eV, high carrier mobility of about 200 cm^2^/(Vs), as well as strong light-matter interaction [121,122].

#### 3.3.2. Optical Properties

**Figure 10:**
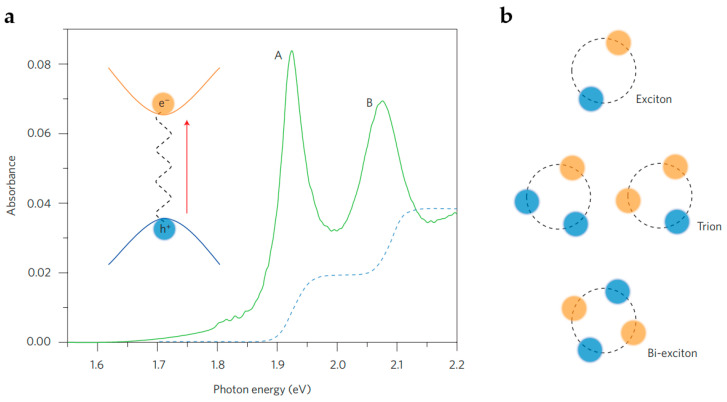
(**a**) Absorption spectrum of monolayer MoS_2_ at 10 K (solid green line). Exciton resonances A and B correspond to the transition of electron–hole pairs from two valence bands split by spin to the conduction band. The absorbance in the absence of excitonic effects is represented by the blue dotted line (in arbitrary units). The inset depicts the Coulomb attraction between the electron–hole pairs generated optically, resulting in the formation of a bound exciton. (**b**) Illustration of excitons and higher-order exciton complexes, including a two-particle neutrally charged exciton, a three-particle charged exciton (trion), and a four-particle double exciton. (Reprinted with permission from ref. [128]. Copyright 2016 Springer Nature Publications).

TMDs are utilized extensively in the fields of photodetection and photoluminescence due to their superior optical characteristics. According to studies, the photoluminescence of bulk MoS_2_ is unremarkable, whereas that of monolayer MoS_2_ is exceptional [123]. Hence, monolayer MoS_2_ can be employed in solar photovoltaic panels, PDs, and photoemitters [124]. Moreover, some members of the TMD family have different bandgaps. Molybdenum and tungsten groups have an optical bandgap in the range of 1~2 eV, which makes them suitable for NIR absorption and emission. The light absorption of monolayer TMDs in the NIR and visible ranges is mostly determined by the direct transition between the conduction and valence band [125,126]. In the absence of excitonic effects, Direct transitions in 2D materials are characterized by a step-function spectrum generated from the energy-independent joint density of states and transition matrix components near the parabolic band edge (Figure 10a) [127,128]. Theoretical estimates have predicted that the exciton binding energy of monolayer TMDs is about 0.5~1 eV [125,129,130]. Meanwhile, higher-order quasiparticles are also observed in the TMDs in addition to excitons (Figure 10b). Currently, triexcitons (bound states of two electrons and one hole or two holes and one electron) have been observed in doped TMDs [131,132], whereas biexcitons (bound states of two excitons) were discovered in monolayer TMDs under pulsed light excitation [133]. Duerloo et al. experimentally demonstrated that mechanical deformation might change the thermodynamic stability of molybdenum and tungsten disulfide monolayers between semiconductors and metallic crystal structures [134]. In addition, they concluded that MoTe_2_ could be an excellent choice for observing phase transitions and modifying the optical response of other materials.

#### 3.3.3. PDs Based on TMDs

As one of the most researched 2D layered materials in photoelectronic devices, TMDs have a bandgap (approximately 1~2 eV) of corresponding wavelengths from visible to NIR ranges, a diverse structure, and stability at room temperature, making them the best choice for PDs that are anticipated to provide excellent responsivity and absorption efficiency. The literature on PDs based on TMDs has continued to grow rapidly in the past few years, including mono-material and hetero-structured devices. The FET and *p*-*n* junction are the most typical device structures in TMD-based PDs.

**Figure 11:**
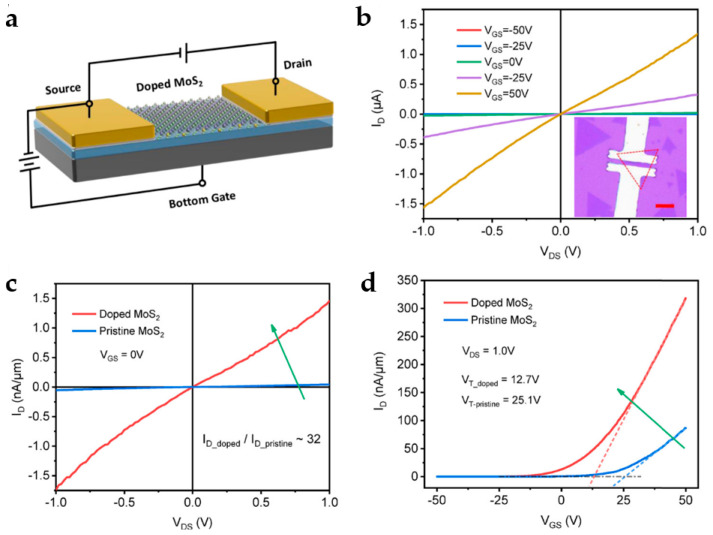
Electrical characteristics of a doped MoS_2_ PD. (**a**) Schematic diagram of the construction of the doped MoS_2_ PD and its electrical connections. (**b**) Output curve of the transistor in dark environment (V_GS_ = −50~50 V). Illustration shows an optical microscope image of a doped MoS_2_ PD with a 10 μm scale (red). (**c**) Output curve (V_GS_ = 0 V) and (**d**) transfer curve of doping and original MoS_2_ FETs in the dark (V_DS_ = 1.0 V). The green arrow shows that the drain current of the FETs based on the doped MOS_2_ is higher than that of the pristine MoS_2_. (Reprinted with permission from ref. [141]. Copyright 2019 American Chemical Society Publications).

MoS_2_ is an attractive candidate for light detection due to its adjustable band gap, high switching ratio, excellent optical properties, high carrier mobility, and stability [135,136,137]. More interestingly, the bandgap varies with the number of layers in the MoS_2_ crystal [138]. Yin et al. produced the first MoS_2_-based PDs in 2011 using a straightforward mechanical exfoliation method, and they investigated the photoelectric properties in detail [139]. Under illumination conditions and a gate voltage of 50 V, the responsivity and response time of the PD is 7.5 mA/W and 50 ms, respectively, which are superior to the first graphene-based PD. Since then, numerous researchers have endeavored to enhance the structure and performance of MoS_2_-based PDs. In 2015, Wang et al. demonstrated a MoS_2_-based PD driven by poly-vinylidene-trifluoride ferroelectric with a detectivity of 2.2 × 10^12^ Jones and a responsivity of 2570 A/W at 635 nm; the light response wavelength was 0.85~1.55 μm [7]. In 2020, Xiao et al. produced a thin MoS_2_ film with excellent thermal stability and superior quality on a SiC substrate using a chemical vapor deposition (CVD) technique [140]. The PDs made of this film exhibit an extraordinarily low dark current of less than 1 nA (at a bias voltage of 20 V), low noise equivalent of 10^−13^~10^−15^ W/Hz^1/2^, and a maximum responsivity of 5.7 A/W under 365 nm UV irradiation. At present, the fundamental obstacle to the implementation of single-layer MoS_2_ in high-performance PDs is the ultrathin structure’s low light absorption, which leads to comparatively poor optical detection performance [141]. Several techniques for synthesizing layered MoS_2_ have been reported, including CVD, chemical and mechanical exfoliation, the Van der Waals epitaxial growth method, and so on. Meanwhile, numerous methods for enhancing the responsiveness of PDs have also been reported, such as heterojunction manufacturing [142] and QD combination [143], etc. However, these technologies have certain limitations that prevent applications from being flexible and miniaturized, including high electron energy consumption, difficult heterojunction production, and insufficient QD stability. The photoresponsivity of MoS_2_-based PDs is normally poor when measured at moderate source-drain voltage (V_DS_) and gate voltage (V_GS_), but it may be enhanced by utilizing the PG effect and increasing the V_DS_, for which higher V_DS_ can extract more photogenerated carriers [139,144,145]. Chemical doping is an efficient method for adjusting Fermi levels and carrier concentrations; it can also improve the optical responsiveness of MoS_2_-based PDs. In 2019, Li et al. proposed a chemical in situ *n*-type doping method to improve the optical response of MoS_2_-based PDs, which was more stable and simpler than the pristine CVD method [141]. Figure 11a depicts the electrical connection and construction of the bottom-gate MoS_2_ PD. The FET’s electrical properties were measured in a dark environment to confirm the difference between the doped and original MoS_2_. The output curve (Figure 11b) shows that drain current (I_D_) and V_DS_ have a good linear relationship, indicating the contact between the electrode and MoS_2_ is an excellent ohmic contact. Figure 11c,d illustrates the distinction of electrical performance between the doped and original MoS_2_ transistors. The doped MoS_2_ transistor had a 32-fold increase in leakage current density, a higher switching ratio, and a higher Fermi level. Overall, the green arrow indicates that doping has a positive effect on the electrical performance of MoS_2_ FETs, as it leads to an increase in drain current. Notably, the responsivity and detectivity of this doped MoS_2_-based PD were 99.9 A/W and 9.4 × 10^12^ Jones at low V_DS_ (0.1 V) and V_GS_ (0 V), respectively, which are 14.6 times and 4.8 times higher than those of the original CVD MoS_2_ PD.

It has been demonstrated that reducing the capture of photogenerated electrons by doping MoS_2_ can enhance the PG effect and device performance. Liu et al. prepared a high-performance PD using carbon QDs and MoS_2_ for the first time [146]. Due to the co-absorption effect and the interlayer exciton transition between MoS_2_ layers and carbon QDs, the performance of the PD is greatly enhanced. The device’s optical responsivity (377 A/W) and detectivity (1.6 × 10^13^ Jones) under 360 nm illumination are 22 and 7 times greater than those of the original MoS_2_ PD, respectively. In 2018, Park et al. fabricated an NIR PD based on multilayer MoS_2_ by chemical exfoliation, which showed a clear light response at 1550 nm by controlling the thickness of the MoS_2_ film [147]. The introduction of Ag nanocrystals improved the responsivity and detectivity to 0.539 mA/W and 0.94 × 10^9^ Jones at 1550 nm, respectively [147]. Pulikodan et al. constructed a PD based on a few-layer MoS_2_ nanosheet in 2020 using a liquid phase stripping approach and systematically explored the light response mechanism [148]. The association between slow-rise current and temperature, as well as vacuum level, in MoS_2_ was proven. In addition, the incorporation of nanoparticles (NPs) can also increase the near-surface electromagnetic field, which in turn results in an enhanced light response. Recently, Zou et al. produced a PD hybrid MXene NPs/MoS_2_ by spin-coating and CVD techniques, showing a high responsivity and detectivity of 20.67 A/W and 5.39 × 10^12^ Jones, respectively, and an external quantum efficiency of 5167% [101]. Local surface plasmon resonance created by MNPs is the reason for the improved performance of this PD.

**Figure 12:**
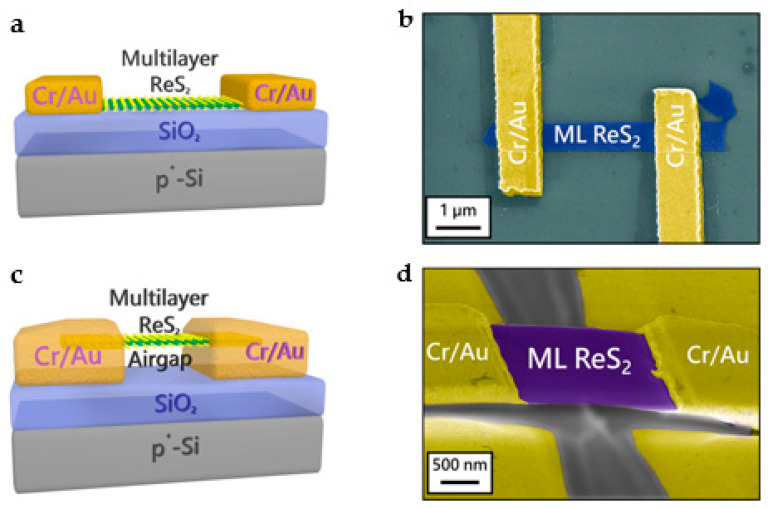
(**a**) Schematic diagram of the supported ReS_2_ transistor (ReS_2_ in contact with the SiO_2_ gate medium) and (**b**) false color SEM image. (**c**) Schematic diagram of a suspended ReS_2_ transistor (with an air gap between ReS_2_ and SiO_2_ gate) and (**d**) false color SEM image (Reprinted with permission from ref. [150]. Copyright 2018, American Chemical Society Publications).

Rhenium disulfide (ReS_2)_ is a promising prospective material in the TMD family for photoelectric detection with an MX_2_ sandwich structure [111]. Due to the distortion of the T phase in ReS_2_ bulk material, its weak interlayer coupling provides ReS_2_ with several remarkable properties, such as the bulk form behaving similarly to the single-layer form in terms of optics, electronics, and vibration [149]. ReS_2_ has a direct bandgap of approximately 1.5 eV. Thakar et al. reported the support and suspension channel FET structures using ReS_2_ as channel material, as shown in Figure 12a–d. Those two structures represent two different trap densities, respectively [150]. They employ gate bias to vary the occupancy of internal and external traps, increasing photocurrent gain while decreasing speed, and the reaction rate is adjusted by more than four orders of magnitude. Methods for adjusting the photoelectric properties of PDs based on low-dimensional materials typically utilize surface charge transfer doping. Nevertheless, previous studies have not systematically explored the relationship between the number of layers and the doping effect. More recently, Zeng et al. investigated different layers of ReS_2_ PDs and demonstrated that doping the top ReS_2_ device with tetrafluorotetracyanoquinodimethane could induce the formation of a vertical *p*-*n* junction [151]. The performance of this device was multiplied by several times compared with the original, and it was discovered that the doping effect is linked to the ReS_2_ thickness. In 2022, Selamneni et al. successfully fabricated a Au-NPs/ReS_2_ device by integrating gold NPs onto the ReS_2_ nanosheet with a responsivity under NIR and visible illumination of ~1.3 and ~2.1 A/W, and a detectivity of 7.27 × 10^11^ and 1.12 × 10^12^ Jones, respectively. Notably, the optical detection performance was 15 times higher than that of the original ReS_2_ device [152]. Effective charge transfer and surface local plasmon resonance at the Au-NPs and ReS_2_ interfaces are two reasons the device’s optical sensing performance improved. These efforts will encourage the development of flexible, high-performance ReS_2_-based PDs for future optoelectronic applications.

In addition to MoS_2_ and ReS_2_, WS_2_ has also emerged as a promising material extensively utilized in photoelectron and PD applications considering its suitable band gap, environmental stability, and high carrier mobility [153,154,155]. For instance, Li et al. first fabricated a flexible PD using WS_2_ prepared by vacuum filtration and hydrothermal intercalation method. The PD can respond to the broadband wavelength of 532~1064 nm, with a responsivity of 4.04 mA/W and a detectivity of 2.55 × 10^9^ Jones under 532 nm irradiation [156]. In addition, this PD has stable light response characteristics under arbitrary bending conditions. In 2021, Kim et al. prepared a PD based on WS_2_ on a flexible substrate by means of electron beam irradiation and radio frequency technology. Its responsivity at wavelength 450, 532, and 635 nm was increased by 1506, 1677, and 1710 times, respectively [157].

### 3.4. Black Phosphorus Based PDs

Black phosphorus (BP), a unique member of 2D layered materials that is the most stable allotrope of phosphorus, was formed by Bridgman in 1914 with a phase transition of white phosphorus (WP) under high pressure [158]. However, BP did not draw much attention for the entire century after its discovery because its quality was difficult to control. Numerous researchers have revealed its unusual photoelectric properties through experiments and theories, thus introducing BP as a 2D layered material with great promise for future electronics and photonics [159,160,161]. BP is a direct bandgap semiconductor with a bandgap range of 0.3 eV (bulk) to 1.7 eV (monolayer), depending on the number of layers [162,163,164], making it more appropriate for optical detection than graphene with a zero bandgap [165]. BP’s bandgap can absorb visible and IR photons, but TMD can only respond to light in the visible. Additionally, BP possesses high light absorption, high room temperature carrier mobility (~5000 cm^2^/(Vs)), and biological compatibility, which make it a candidate to apply in various devices, such as PDs, ultrafast lasers, optical switching, modulators, sensors, and even biomedicine. Due to its distinctive puckered structures that produce anisotropic in-plane characteristics, it is a superb candidate for researching novel physical processes as well.

#### 3.4.1. Morphology and Structure

**Figure 13:**
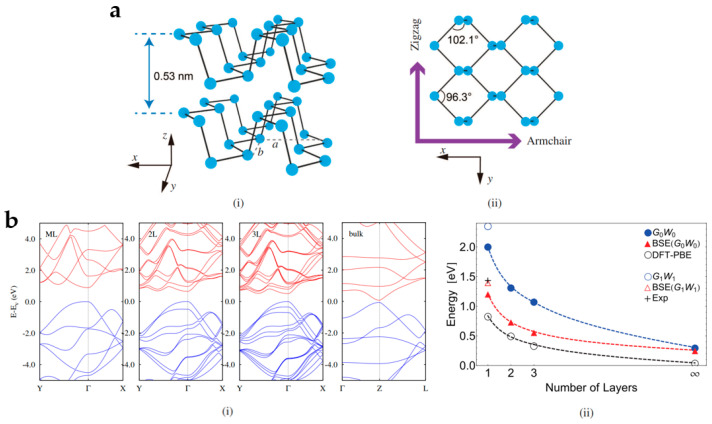
Crystal structure of BP. (**ai**) Perspective of the crystal structure of BP, the interlayer spacing was 0.53 nm. (**aii**) Top view of monolayer BP, where x and y correspond to armchair and zigzag directions, respectively (Reprinted with permission from ref. [166]. Copyright 2021 Springer Nature Publications). (**bi**) The band structures of one-, two-, three-layer and bulk phosphorus were calculated using density functional theory. (Reprinted with permission from ref. [163]. Copyright 2014 American Physical Society Publications). (**bii**) The relationship between the band gap and the number of layers in theory and experiment. (Reprinted with permission from ref. [160]. Copyright 2014 American Physical Society Publications).

BP is converted from WP under high pressure. Therefore, its crystal structure is similar to WP [166]. Figure 13a depicts the crystal structure of BP, which consists of four P atoms, each of which combines with three adjacent P atoms via sp^3^ orbitals to form two unequal orientations, parallel and perpendicular to the atomic ridge corresponding to armchair and zigzag directions, respectively. Compared with WP, BP exhibits relatively better stability owing to its orthogonal crystal structure. Many monolayers of BP are stacked into bulk BP by weak van der Waals interactions, with tetrahedral structural units and sp^3^ hybridizing each other to form each successive layer, resulting in a non-planar folded hexagonal structure similar to the folded honeycomb structure (Figure 13(ai)). Typically, different bond angles in BP result in various bond lengths. One is the in-plane bond with a bond length of 0.2224 nm, the other is the external plane bond connecting the top and bottom P atoms with a bond length of 0.2244 nm. The bond length of the interlayer P-P in the block BP is 0.55 nm, indicating that the layers of BP are held together by weak van der Waals forces rather than bond interactions. Figure 13(aii) shows the top view of monolayer BP. The unit consisted of four P atoms in BP joined to create continuous layers by breaking down the individual bonds to generate sp^3^ hybridization, with bond angles of 96.300° and 102.095° that approach 102.1° for a perfect tetragonal [167,168], offering better crystal network stability [15,169]. Figure 13b illustrates BP’s band structure, revealing that both monolayer and bulk BP have direct bandgaps. As the number of layers rises, the bandgap redshifts from 2.0 eV to 0.3 eV (Figure 13(bi)) [166]. In 2016, Feng et al. examined the bandgaps of BP with various thicknesses using absorption spectroscopy. They found that the monolayer, bilayer, and block BP bandgaps were 1.73 eV, 1.15 eV, and 0.35 eV, respectively [170]. Although the bandgap of BP varies with thickness, it always retains the features of direct bandgap [171], which complements the zero bandgap of graphene and the narrow bandgap of TMDs [172]. Figure 13(bii) depicts the relationship between the bandgap and the number of layers. Notably, the Fermi energy level moves toward the valence band as the thickness increases, resulting in a *p*-type characteristic usually evident in BP-based devices.

#### 3.4.2. Optical Properties

**Figure 14:**
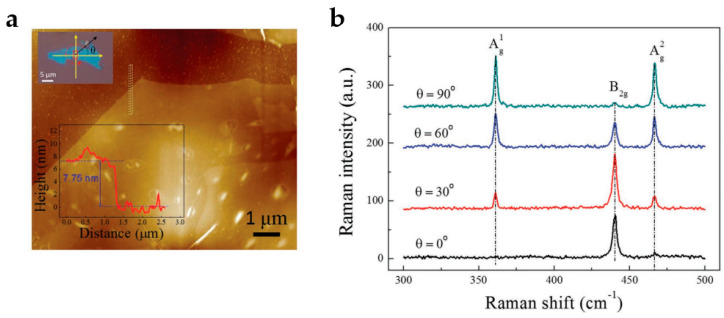
The optical properties of BP. (**a**) An AFM image of a thin BP flake reveals an approximate thickness of 7.75 nm. Inset: a photograph of this BP flake. (**b**) Raman spectrum of BP utilizing polarized laser stimulation in various orientations. (Reprinted with permission from ref. [173]. Copyright 2016 John Wiley and Sons Publications).

In many aspects, BP is more attractive than graphene and other materials thanks to its unique light response and strong anisotropy. Figure 14a represents the optical image of BP. Experiments and theories have confirmed that few-layer BP exhibits a moderate-intensity photoluminescence peak, while the light luminescence peak of monolayer BP can reach 1.45 eV [167]. The atomic force microscope (AFM) image of a BP flake is shown in Figure 14a. The Raman peaks at 365, 440, and 470 cm^−1^ correspond to $A_{g}^{1}$, $B_{g}^{2}$, and $A_{g}^{2}$ of the BP vibration modes, respectively [173,174], as depicted in Figure 14b. As the polarization of the excitation laser progressively increases from 0 to 90°, the fundamental atomic vibration of the $B_{g}^{2}$ mode is in the x direction; hence, the $B_{g}^{2}$ mode’s intensity decreases substantially. Moreover, Raman observations of single-, double-, and bulk BP indicate that Raman peaks in monolayer BP are redshifted [159]. When BP flakes are exposed to polarized light in the z direction with a range of 0°~300° and a step size of 30°, all polarization directions of the IR spectrum exhibit a notable increase at 2400 cm^−1^, corresponding to a bandgap of 0.3 eV [15]. The bandgap of BP is the most significant factor in determining optical absorption. The band structure will determine the optical properties of 2D materials, particularly those that may interact with light. Furthermore, with appropriate polymer functionalization, the optoelectronic characteristics of BP-based nanocomposites can be improved, enabling their utilization in nonlinear optical properties, electrical and optoelectronic devices, and biomarker detection.

#### 3.4.3. PDs Based on BP

In recent years, BP has shown great application potential in the field of PD, especially NIR detection, due to its marvelous properties such as a modest bandgap (0.3~2 eV), high carrier mobility (1000 cm^2^/(Vs)), considerable switching ratio (up to 10^6^), and anisotropy. Buscema et al. developed a BP-based FET with a responsivity of 4.8 mA/W to 940 nm NIR light and a response time of less than 4 ms, significantly superior to that of the WS_2_ device [175]. Engel et al. developed an array of optical detectors that could be used for broadband imaging, demonstrating the enormous potential of BP in optoelectronic detection and display [176]. However, due to the easy oxidation features of BP, it is easy to produce surface contamination during the device manufacturing process using conventional lithography, which severely limits its practical application. Therefore, it is essential to explore better device preparation schemes and introduce defects effectively to improve the performance of BP-based PDs. To date, a variety of approaches have been implemented to improve BP’s environmental stability. For example, BP compounds were synthesized using wet chemistry with electron-poor and polarimetric polycyclic aromatic hydrocarbons, resulting in strong non-covalent interactions between BP and molecules. In this regard, the physical encapsulation and chemical passivation prior to use is of great significance. Zhang et al. used hydrophobic polyionic liquid poly hexafluorophosphate (PIL-TFSI) to encapsulate BP QDs for PDs and extensively investigated the morphology, composition, and characteristics of BP-PIL in 2019 [177]. The results suggested that the unique hydrophobic properties of PIL-TFSI and the fluoridation of BP QDs can considerably enhance the environmental stability of BP QDs. Compared with the conventional BP-based PDs, the BP-PIL-based PDs have better optical response characteristics and longer-term environmental stability. In addition, this self-healing PD exhibited a distinct on/off signal after 50 cycles, indicating the immense practical potential of BP-PIL-based PDs. Subsequently, Fan et al. proposed a PD constructed from chemically modified BP sheets that showed high performance and environmental stability over 4 months [178].

In addition, the poor light absorption of BP limits its utilization in high-performance PDs. To address this issue, heterostructures are used as hybrid structures to trap electron–hole pairs efficiently. Early publications have shown that QDs exhibit remarkable localized photon trapping capabilities owing to quantum confinement and surface effects and are considered superior light adsorbents to enhance the performance of PDs. Kwak et al. made the first 0D-2D hybrid PD using InP QDs and BP, which exhibit the responsivity and detectivity of 1 × 10^9^ A/W and 4.5 × 10^16^ Jones, respectively, under 405 nm illumination [178]. The exceptional performance of this hybrid PD is a result of photogenerated electron injection from the InP QD into the BP. In 2020, Qiao et al. used a liquid separation method to prepare BP QDs and built heterojunction PD [179]. The quantum confinement effect of BP QDs and their synergistic effect with MoS_2_ nanosheets significantly improved the optical response of the device. The PD’s optical response at 0.6 V bias and the photocurrent at zero bias are approximately 2.8 times and 2.2 times that of the original MoS_2_-based PD, respectively, indicating its remarkable self-powered PD properties. These strategies improve the responsivity, optical gain, and response time to some extent but require managing toxic substances, making them challenging to implement on a large scale.

Local surface plasmon resonance (LSPR) is a technique to improve the light absorption of materials used with noble metal NPs containing many free electrons, such as Au, Ag, and Pt. Light can be trapped on the surface of metal NPs using LSPR, resulting in enhanced photoabsorption from visible to IR wavelengths. Jeon et al. created a PD based on BP/Au NPs using the LSPR method, which significantly improved the PD’s performance [180]. The density of NPs can be adjusted by simply depositing Au NPs on the BP surface and regulating the annealing process. Due to the integration of Au NPs on the BP channel, the photoabsorption is enhanced while the dark current is suppressed. The laser responsivity of visible and IR wavelengths is increased to 6000 and 500 A/W, respectively. In 2021, Tian et al. published a method of integrating high-performance BP-based PD on silicon planar photonic crystal cavities, in which the light absorption of BP is greatly enhanced due to the interaction between light and matter in the cavity [181]. Thanks to the relatively short BP channel, the PD has a responsivity of 125 mA/W and a dark current of less than 20 nA at 0.5 V bias voltage. Recently, Cao et al. built a van der Waals heterostructure based on BP with a 2D chiral perovskite for the first time [182]. The advantages of BP in IR photoelectrons combined with the effective photoabsorption and charge transfer of perovskites in this straightforward heterostructure make the PD’s responsivity and optical gain increase by several orders of magnitude compared with BP alone.

### 3.5. Hexagonal Boron Nitride (hBN) Based PDs

#### 3.5.1. Morphology and Structure

**Figure 15:**
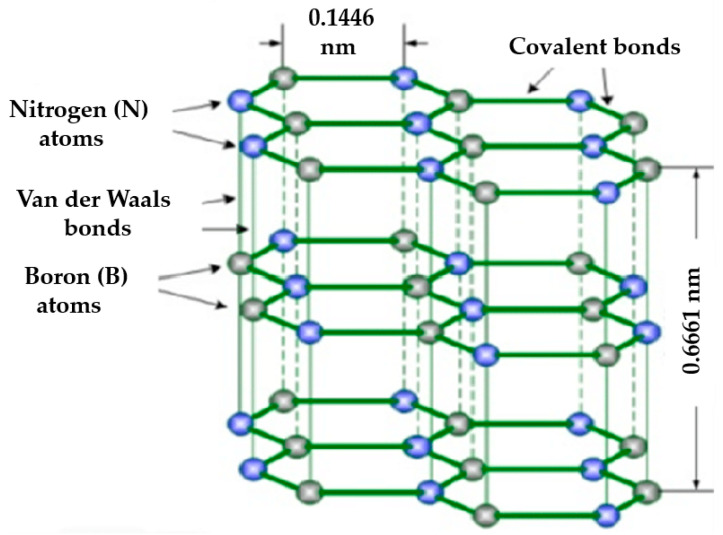
Schematic of hBN structure. (Reprinted with permission from ref. [183]. Copyright 2012 SPIE Publications).

hBN is regarded as “white graphene” due to its similarity in crystal structure to graphite. However, unlike graphite, whose layers are stacked using the AA method, hBN employs the ABAB method, with nitrogen and boron atoms placed alternately. As illustrated in Figure 15 [183], the interaction of van der Waals forces and strong ionic bonds between the layers makes the structure of hBN more stable. Monolayer hBN has a 2D honeycomb structure similar to graphene to form sp^2^ hybridized B–N bonds. Although they have similar structures, the electrical properties of the two types of 2D materials are different. Graphene has a zero bandgap, whereas monolayer hBN has a bandgap of 5.97 eV [184,185]. Since hBN layers are coupled by weak van der Waals forces, few-layer or even monolayer hBN can be easily obtained by the mechanical exfoliation of bulk monocrystals [186]. Although hBN has poor intrinsic conductivity, it can be employed in electrocatalysis through structural and electronic modifications. In addition, it exhibits high specific surface area, numerous active centers, strong thermal stability, and excellent mechanical strength [187,188].

#### 3.5.2. Optical Properties

**Figure 16:**
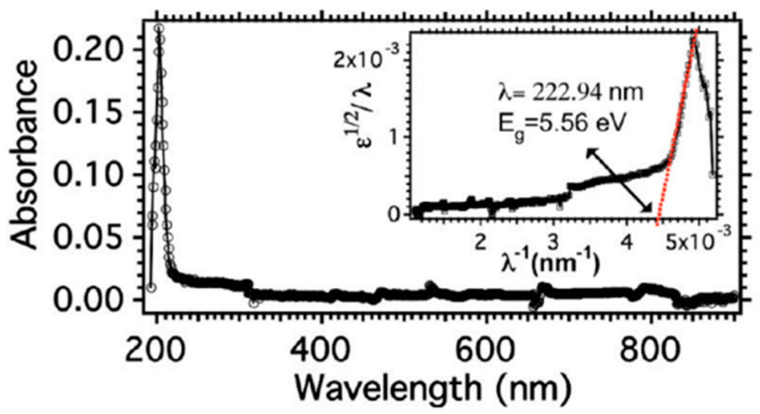
UV-visible absorption spectra of hBN films at room temperature. (Reprinted with permission from ref. [189]. Copyright 2010 American Chemical Society Publications).

As shown in Figure 16 [189], hBN has high transparency in the range of 250~900 nm, with a transmittance of 99% and a strong absorption peak in the DUV region (200~220 nm). Calculations reveal that the bandgap of multilayer hBN is 5.56 eV [189], whereas the monolayer hBN and bulk hBN are approximately 5.84~6.07 eV [190] and 5.2 eV [191], respectively. The interaction between the hBN layers leads to an increase in electron band dispersion and a corresponding decrease in the band gap. hBN samples can exhibit DUV or UV luminescence through electron beam excitation. The existence of defects or lattice defects results in several extra exciton peaks in the cathodic luminescence spectrum [192]. Because of its broad bandgap and UV emission capabilities, hBN has broad application prospects in UV lasers [193], photon emission [194], and DUV detectors [195]. Furthermore, hBN has excellent nonlinear optical properties such as two-photon absorption, deep penetration, and high three-dimensional resolution, making it ideal for multi-photon imaging optical applications.

#### 3.5.3. PDs Based on hBN

With its ultra-wide bandgap of approximately 6 eV, hBN is highly transparent to visible light and capable of absorbing DUV light due to its inherent absorption properties. Its high band edge absorption coefficient also makes it an ideal choice for DUV photoelectric detection, without requiring any additional processing or doping. This makes hBN a promising material for a wide range of applications in the field of optoelectronics, including DUV photodetection, UV-light-emitting devices, and DUV light sources [196]. Moreover, it can operate in harsh environments and high temperatures because of its excellent oxidation resistance, high temperature resistance, and corrosion resistance [188,197]. In addition, hBN has an exceptionally large bandgap and, thus, does not require a solar filter or an extra cooling system, greatly simplifying device design [196,198]. Recently, numerous scientists have explored the photoelectric performance of hBN-based PDs. Gao et al. fabricated high-quality hBN thin films on a sapphire substrate using the ion beam sputtering deposition in 2019 [199]. Compared with transferred hBN, the DUV hBN-based PD prepared by this method has better performance. For instance, the on/off ratio can reach 6800, the relative detectivity exceeds 1.8 × 10^10^ Jones, and the response time is about 1 ms. High quality hBN crystals were prepared by Zhang et al. at atmospheric pressure by using the flux growth method [200]. Two types of solar-blind PD with top contact electrode and bottom contact electrode were fabricated on the basis of this hBN layer, which was mechanically stripped at 15–17 nm. The results demonstrate that the device has a specific detectivity of 3.68 × 10^8^ Jones at 215 nm. Reports of hBN-based PDs primarily fabricated on rigid substrates exhibit low optical response, and little about applications in flexible electrons. In 2021, Veeralingam et al. deposited hBN nanosheets on Cu (111) substrates to make DUV PDs with superior performance. The ultra-high responsivity, specific detectivity and external quantum efficiency were 5.022 A/W, 6.1 × 10^12^ Jones, and 2945%, respectively [197]. Wu et al. fabricated a graphene/hBN/*n*-AlGaN DUV PD in 2020 [201]. AlGaN semiconductors with a large bandgap can discern DUV signals without UV filters. Moreover, hBN insulators are excellent for decreasing dark current and enabling photogenerated carrier quantum tunneling. By reducing the strain issue between graphene and conventional bulk insulators, nanographene-hBN heterostructures can boost the performance of PDs.

## 4. Conclusions

This paper gives a comprehensive summary on the recent research progress of PDs based on 2D materials including graphene, MXenes, TMD, BP, and hBN, with emphasis on their morphology and structure, optical properties, as well as detailed applications in PDs. The past decade has witnessed tremendous progress and interest in emerging PDs built from 2D materials, and the key to the success is their unique structural, electrical, optical, mechanical, and thermal properties. The 2D material-based PDs have shown applications in broadband detection, high sensitivity detection, polarization sensitive light detection, and so on. However, there are still some challenges in achieving high-performance PDs, such as growing high-quality 2D materials, achieving higher quantum efficiency, and effectively separating the photogenerated electron–hole pairs. Several strategies have been demonstrated to effectively address those issues: (1) create devices with new architectures such as grating and antennas to enhance the interaction between optical and 2D materials; (2) improve the synthesis technique to produce high-quality 2D materials; and (3) employ surface encapsulation or doping, modification, and other techniques to enhance the performance of PDs. In a word, to fully harness the features of 2D materials, further work is required to comprehend their pristine characteristics and the physical process that dominates photodetection. 

## 5. Outlook

PDs based on 2D materials show great potential for future development and application in various fields. Firstly, due to their unique properties, such as large surface-to-volume ratio and high carrier mobility, 2D material-based PDs may achieve higher sensitivity and resolution than traditional detectors. Secondly, 2D material-based PDs may offer wider spectral ranges due to their tunable bandgap and broad absorption spectrum, which may lead to new applications in the areas of spectroscopy, astronomy, and telecommunications. Moreover, the ultrathin nature of 2D materials may allow for lower power consumption and smaller size, as well as compatibility with flexible and transparent substrates. These advantages can enable applications in wearable devices, Internet of Things (IoT), and other portable electronics. Finally, PDs based on 2D materials may be integrated with other devices, such as transistors, memory cells, and sensors, to create multifunctional systems. Overall, the continued progress in the field of 2D material-based PDs holds great promise for a wide range of applications in fields such as communications, sensing, and imaging, and it is expected to have a significant impact on many areas of science and technology. We believe that 2D materials will be extensively employed in the optoelectronic field in the near future through improving device design and material engineering.

## Figures and Tables

**Table 1:** PDs based on graphene.

Device Structure	Detectivity	Response Time	Responsivity (A/W)	Ref.
Graphene/Si/SiO_2_ PD		1 μs	0.1 A/W	[57]
Graphene/HfO_2_/a-MoS_2_ PD		68 μs	5.36 A/W (473~2712 nm)	[62]
WSe_2_–graphene–MoTe_2_ PD	1.21 × 10^11^ Jones (0 V bias)	468/428 μs (rise/decay)	40.84 mA/W (550 nm)	[63]
Graphene/Si/graphene oxide PD		1 ms	0.65 A/W (633 nm)	[64]
Graphene/P-InP PD	1.3 × 10^10^ Jones		5.2 mA/W (808 nm)	[65]
Graphene nanofilms/Si PD		371 ns	0.4 mA/W (1870 nm) 10 A/W (after avalanche)	[66]
Graphene/Ge PD	9.6 × 10^9^ Jones		1.27 A/W (1550 nm)	[67]
multilayer graphene/InSe PD		22 ms	1.88 × 10^5^ A/W	[68]
Graphene/Si/Gd_3_Fe_5_O_12_ PD	1.35 × 10^13^ Jones (633 nm)	0.15 ms	0.9 A/W	[69]
Graphene/Pbs PD	10^9^ Jones (1200 nm)		10^4^ A/W	[70]
Graphene/TiO_2_ films/PbS PD	1.5 × 10^12^ Jones (1 V bias)	35 ms	1.2 × 10^4^ A/W (635 nm)	[71]
Graphene nanofilm/silicon Heterojunction PD	1.6 × 10^11^~1.9 × 10^9^ Jones	20~30 ns	3~11 mA/W	[72]
MoS_2_/graphene/GaAs PD	4.86 × 10^10^ Jones	46.8 μs	19.9 mA/W (808 nm)	[73]
Tellurium/Graphene PD	1.04 × 10^9^ Jones (2 μm)	28 μs	96.4 mA/W	[74]
Graphene/germanium hybrid	5.28 × 10^10^ Jones		2.02 A/W	[75]

**Table 2:** PDs based on MXenes.

Device Structure	Response Wavelength	Detectivity	I_light_/I_dark_ Ratio	Responsivity (A/W)	Reference
InSe/Ti_2_CT_x_ avalanche PD	0.4–1.55 μm	7.3 × 10^12^ Jones		1.0 × 10^5^ A/W	[90]
Ti_3_C_2_T_x_-TiO_2_ photodetector	405 nm	8.40 × 10^4^ jones		0.078 mA/W	[91]
Ti_3_C_2_T_x_/GaAs Schottky junction	405–980 nm	~1.23 × 10^13^ Jones	5.6 × 10^5^	~1.46 A/W	[92]
Ti_3_C_2_T_x_/TiO_2_ heterojunctions	280–400 nm			2.06 mA/W	[93]
MXenes–β-Ga_2_O_3_ Schottky junctions	248 nm	6.1 × 10^12^ Jones	1.6 × 10^4^	12.2 mA/W	[94]
ZnO/Ti_3_C_2_T_X_/ZnO Schottky PD	254 nm	2.53 × 10^9^ Jones		6.17 × 10^−2^ A/W	[95]
Mxene-GaAs-Mxene PD	532, 780, 830 nm	11.6 × 10^10^ Jones		278 mA/W	[96]
Perovskite/MXene-Based PD	450 nm	6.4 × 10^8^ Jones	2.3 × 10^3^	44.9 mA/W	[97]
Mo_2_C/MoGeSiN_4_ hot-electron PD	1550 nm			176 mA/W	[98]
MXene-embedded transparent PD	365 nm	4.1 × 10^10^ Jones		20 mA/W	[99]
Ti_3_C_2_T_x_ MXene/Si based PD	980 nm	5.4 × 10^13^ Jones		302 mA/W	[100]
MXene/MoS_2_ PD	635 nm	5.39 × 10^12^ Jones		20.67 A/W	[101]
ZnO QD/MXene nanoflake PD	350 nm	7.1 × 10^11^ jones		425 mA/W	[102]
Co-CoO_x_/NC/Mo_2_CT_x_ heterostructure PD	350, 400, 450, 550, and 650 nm	4.5 × 10^7^ Jones		20.7 μA/W	[103]

## Data Availability

No new data were created or analyzed in this study. Data sharing is not applicable to this article.
